# Characterization of two rat models of cystic fibrosis—KO and F508del CFTR—Generated by Crispr‐Cas9

**DOI:** 10.1002/ame2.12091

**Published:** 2019-11-25

**Authors:** Elise Dreano, Marc Bacchetta, Juliette Simonin, Louise Galmiche, Claire Usal, Lotfi Slimani, Jérémy Sadoine, Laurent Tesson, Ignacio Anegon, Jean‐Paul Concordet, Aurélie Hatton, Lucile Vignaud, Danielle Tondelier, Isabelle Sermet‐Gaudelus, Marc Chanson, Charles‐Henry Cottart

**Affiliations:** ^1^ INSERM 1151 INEM Université de Paris Paris France; ^2^ Département de Pédiatrie Gynécologie & Obstétrique et Département de Physiologie Cellulaire & Métabolisme Université de Genève Genève Switzerland; ^3^ Département de Pathologie APHP CHU Necker‐Enfants Malades Paris France; ^4^ Centre de Recherche en Transplantation & Immunologie UMR 1064 INSERM Université de Nantes Nantes France; ^5^ Plateforme Trangénèse Rat & ImmunoPhénomique INSERM 1064 & SFR François Bonamy CNRS UMS3556 Nantes France; ^6^ Pathologie, Imagerie & Biothérapies Orofaciales Montrouge France; ^7^ Plateforme Imageries du vivant Faculté de chirurgie dentaire Université de Paris Paris France; ^8^ INSERM U1154 CNRS UMR7196 MNHN TACGENE Paris France; ^9^ AP‐HP Centre Maladie Rare Mucoviscidose et Maladies du CFTR Assistance Publique Hôpitaux de Paris Hôpital Necker‐Enfants Malades Paris France; ^10^ Faculté de Médecine de Paris Université de Paris Paris France; ^11^ Faculté de Pharmacie de Paris Université de Paris Paris France

**Keywords:** animal models, CFTR channel activity, CFTR modulators, cystic fibrosis, primary cultures, rat

## Abstract

**Background:**

Genetically engineered animals are essential for gaining a proper understanding of the disease mechanisms of cystic fibrosis (CF). The rat is a relevant laboratory model for CF because of its zootechnical capacity, size, and airway characteristics, including the presence of submucosal glands.

**Methods:**

We describe the generation of a CF rat model (F508del) homozygous for the p.Phe508del mutation in the transmembrane conductance regulator (*Cftr*) gene. This model was compared to new *Cftr* ^−/−^ rats (CFTR KO). Target organs in CF were examined by histological staining of tissue sections and tooth enamel was quantified by micro‐computed tomography. The activity of CFTR was evaluated by nasal potential difference (NPD) and short‐circuit current measurements. The effect of VX‐809 and VX‐770 was analyzed on nasal epithelial primary cell cultures from F508del rats.

**Results:**

Both newborn F508del and Knock out (KO) animals developed intestinal obstruction that could be partly compensated by special diet combined with an osmotic laxative. The two rat models exhibited CF phenotypic anomalies such as *vas deferens* agenesis and tooth enamel defects. Histology of the intestine, pancreas, liver, and lungs was normal. Absence of CFTR function in KO rats was confirmed ex vivo by short‐circuit current measurements on colon mucosae and in vivo by NPD, whereas residual CFTR activity was observed in F508del rats. Exposure of F508del CFTR nasal primary cultures to a combination of VX‐809 and VX‐770 improved CFTR‐mediated Cl^−^ transport.

**Conclusions:**

The F508del rats reproduce the phenotypes observed in CFTR KO animals and represent a novel resource to advance the development of CF therapeutics.

## 1 INTRODUCTION

Genetically engineered animals are essential for gaining a proper understanding of cystic fibrosis (CF) and developing new therapies. Five animal models (mouse, rat, ferret, pig, and sheep) that lack functional CF transmembrane conductance regulator (CFTR) channels have been developed.^1^ The CF mouse has provided a considerable amount of information on the physiopathology of CFTR defects at the organ level, but its main caveat is the lack of a spontaneous lung phenotype.^2^, ^3^ One of the reasons for this is the lack of submucosal glands (SMGs), which express CFTR at a high level in humans and other animal models such as the ferret and pig.^4^ Indeed, these two animal models better reproduce the CF phenotypes observed in humans, from inflammatory and infectious lung disease to in utero meconium ileus and CF‐related diabetes.^5^, ^6^ However, the ferret, sheep, and pig models are resource‐intensive and pose a number of challenges that limit their widespread use for translational research.

New animal models that develop pulmonary disease and are less challenging in terms of cost and maintenance are required. Due to its zootechnical capacity, size and airway characteristics, the rat may represent a valuable animal model to explore CF.^7^ Tuggle et al, using zinc‐finger endonuclease technology, recently developed a CFTR^−/−^ ‐ Knock out (KO) ‐ rat model that reproduces many aspects of human disease, including growth failure, tooth enamel abnormalities and bone disease.^8^, ^9^ Interestingly, the CFTR^−/−^ rats developed mucus defects with age leading to delayed mucociliary clearance.^10^ This is related to the fact that rats have SMGs throughout the cartilaginous tracheal airways that develop postnatally.^11^ Furthermore, early reports of *CFTR* silencing using antisense delivered in utero with adenoviruses resulted in the development of pulmonary disease in newborn rats. CF‐associated lung diseases including fibrosis and chronic inflammation became apparent in adult rats at 3.5 months of age following in utero antisense CFTR treatment.^12^, ^13^ These data suggest that the rat may represent a useful model to provide further insights into the physiopathology of CF lung disease.

In the expanding area of novel modulators, whose clinical efficiency is based on in vitro assays in human bronchial primary cells, it is crucial to assess the efficacy of mutant CFTR correctors at the organism level in animal models, including rats. Since the most common *CFTR* mutation in humans is deletion of phenylalanine at position 508, we generated a rat model homozygous for this mutation (p.Phe508del *Cftr*, *Cftr* ^F508del/F508del^, F508del thereafter) using the Clustered Regularly Interspaced Short Palindromic Repeats ‐ CRISPR associated protein 9 (CRISPR‐Cas9) strategy. This model was compared to new *Cftr* ^−/−^ rats (CFTR KO thereafter) based on the main histological features and electrophysiological characteristics.

## 2 METHODS

### 2.1 Generation of F508del and CFTR KO rats

All animal care and procedures performed in this study were approved by the Animal Experimentation Ethics Committee of the Pays de la Loire region, France, in accordance with the guidelines of the French National Research Council for the Care and Use of Laboratory Animals (Permit Numbers: CEEA‐PdL‐2015‐692). All animal care and procedures performed in the Paris facility were approved by the Animal Experimentation Ethics Committee of Paris Descartes University and were registered with ministerial numbers APAFiS #9462 and APAFiS #13755. All animal studies performed in Geneva were approved by the Swiss Federal Veterinary Office and were in accordance with the established Swiss guidelines and regulations. The procedures are described in detail as supplementary data elsewhere in the article.^14^, ^15^ Rats were generated using CRISPR‐Cas9 technology and genome editing techniques for the introduction into the genome of single‐stranded oligo donor sequences by microinjection into rat zygotes. For the F508del rat, the target sites were based on exon 12; donor DNA generated a codon deletion at F508 and the creation of one NdeI restriction site. For the CFTR KO rat, the target sites were based on exon 3 (according to rNO5 6.0 nomenclature); donor DNA generated a frameshift and the creation of one XbaI restriction site and a premature stop codon. This led to generating two CFTR KO founders (referred to as MUKORATs 8.3 and 6.4). Because no phenotype differences were observed between 8.3 and 6.4 MUKORATs, data obtained from these animals were pooled.

### 2.2 Animal husbandry of F508del and CFTR KO rats

*CFTR^+/F508del^* and *CFTR* ^+/−^ rats were inbred to generate F508del and CFTR KO animals, respectively. Laxative (PEG 3350‐KleanPrep; Norgine Pharma or Transipeg; Mundipharma) was added to the drinking water. To reduce mortality, F508del and CFTR KO were fed after weaning with a liquid diet composed of DietGel 31M and Boost (Clear H_2_O^®^, Ssniff‐Spezialdiäten GmbH) and Dietgel 31M respectively as their respective *CFTR^+/+^* littermates. Because of fatal growth retard, the diet of CFTR KO rats and littermates, ground chow mixed with laxative‐containing water (DietGel + laxative), was progressively introduced in the diet for F508del and KO rats. Detailed information is in the Supporting Information.

### 2.3 Characterization of F508del and CFTR KO rats

The colon, ileum, lung, pancreas, and liver from F508del and CFTR KO rats were dissected, fixed, and embedded in paraffin. Blocs were cut into 4‐5 µm sections, stained with hematoxylin and eosin (H&E) or periodic acid Schiff (PAS) and examined by light microscopy. Additional staining with Alcian blue to reveal mucus‐secreting cells was also performed on intestinal and lung tissue of CFTR KO rats.

Mandibles from 8‐ to 14‐week‐old littermate wild‐type (WT) and F508del or 14‐ to 37‐week‐old CFTR KO rats were scanned by high‐resolution X‐ray micro‐computed tomography (CT) and total enamel volume and density were quantified. The detailed protocol is provided in the Supporting Information.

### 2.4 Primary nasal cell cultures

The detailed protocol for nasal epithelial cell culture is provided in the Supporting Information. Briefly, the nasal mucosa was scraped and subjected to protease digestion. The cell suspension was then filtered, centrifuged, and the pellet suspended in growth medium for cell expansion on 75‐cm^2^ flasks. Cells were then trypsinized, seeded on Transwell filters, and differentiated at an air‐liquid interface for 20‐30 days.

### 2.5 Nasal potential difference

Transepithelial potential (*V*_TE_) was measured between an Ag/Ag reference electrode and an Ag/AgCl exploring electrode, as described previously with minor adaptations.^16^ The following parameters were recorded during *V*_TE_ measurements: (a) negative stable baseline during Cl^−^ solution perfusion (Baseline *V*_TE_) and (b) sequential *V*_TE_ change in response to the addition of amiloride (∆Amiloride), low Cl^−^ plus forskolin (∆Low Cl^−^ + FK), and Inh‐172 plus GlyH‐101 (∆Inh‐172 + GlyH‐101). The detailed protocol is provided in the Supporting Information.

### 2.6 Short‐circuit currents

Cystic fibrosis transmembrane conductance regulator channel activity was assessed by the measurement of transepithelial short‐circuit currents (*I*_sc_) in Ussing chambers (Physiologic Instruments). The following parameters were recorded: (a) stable baseline (Baseline *I*_sc_) and (b) sequential *I*_sc_ change in response to the addition of amiloride (∆Amiloride), IBMX plus forskolin (∆IBMX/FK), GlyH‐101 or Inh‐172 (ΔInh‐172), and bumetanide (∆Bumetanide). The detailed protocol is provided in the Supporting Information.

### 2.7 Statistics

Data are expressed as means ± SEM calculated using the Prism software package (GraphPad Prism® v6). The Wilcoxon paired signed‐rank test was used to evaluate treatment (VX‐770 or/and VX‐809) effect. Between‐group comparisons were evaluated using the Mann‐Whitney test. N represents the number of rats and n represents the number of filters.

## 3 RESULTS

### 3.1 Generation of F508del and KO rats

**Figure 1:**
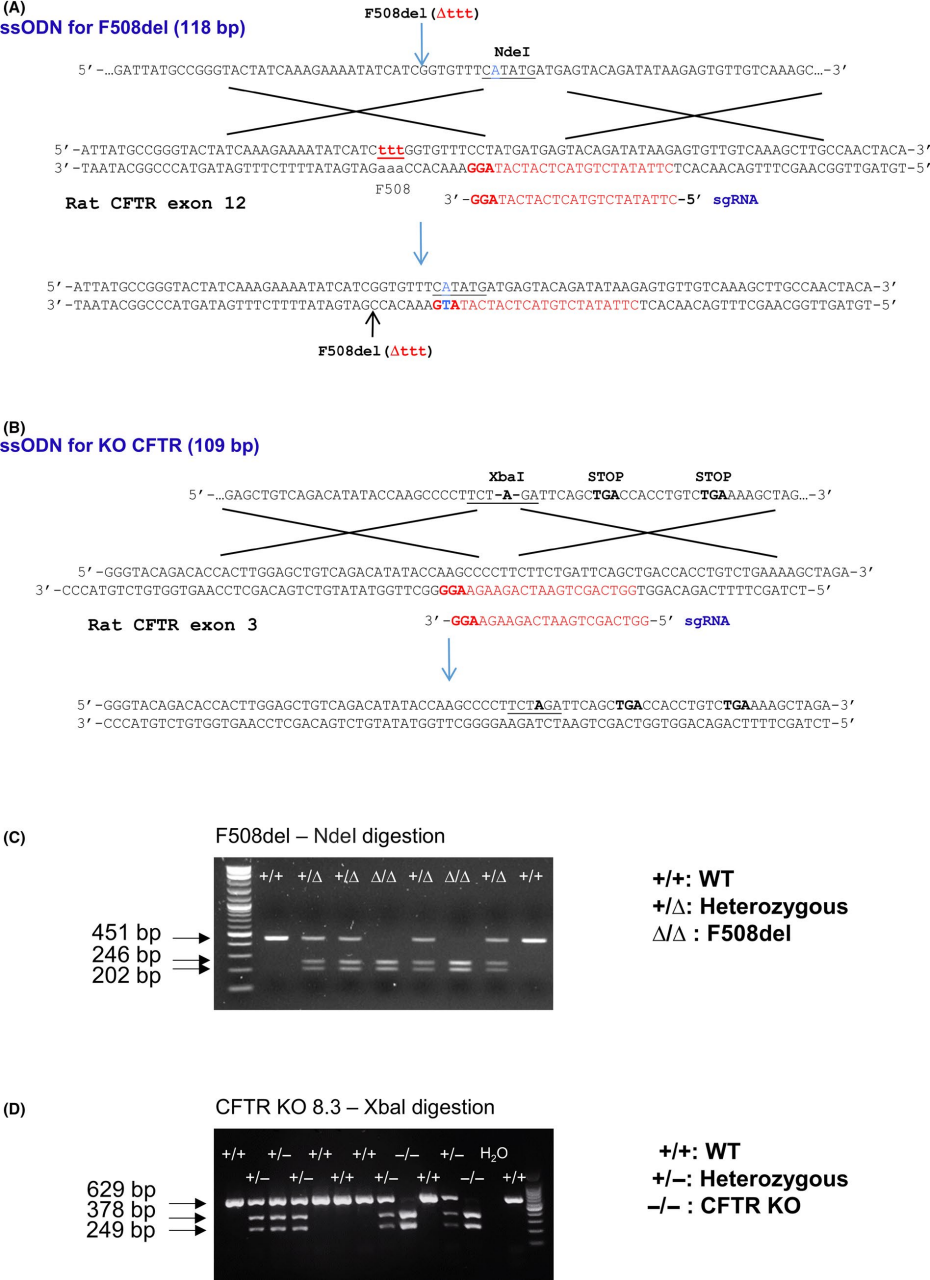
CRISPR strategy. A, For F508del rats, single‐guide RNA (sgRNA) was designed to target exon 12 and the F508 deletion was driven via Knock In (KI) with a ssODN including the ttt deletion, homology arms, and a restriction site to facilitate the genotyping. In blue, a mutation to cancel the sgRNA Protospacer Adjacent Motif (PAM). B, For cystic fibrosis transmembrane conductance regulator (CFTR) KO rats, sgRNA was designed to target exon 3 and the KO was driven via KI with a ssODN including homology arms, a restriction site to facilitate the genotyping and two STOP codons. C and D, Animal genotypes. Representative PCR genotyping for F508del with *Nde*I digestion (C) and CFTR KO with *Xba*I digestion (D) rat generation. WT, wild‐type

Cystic fibrosis transmembrane conductance regulator F508del and KO rats were generated by delivering simultaneously, into one‐cell stage zygotes, specific single‐guide RNA (sgRNA) and ssODN designed to introduce mutations at codon 508 in exon 12 (insertion of NdeI recognition site) and in exon 3 (insertion of XbaI recognition site) of *Cftr* gene, respectively (Figure 1A,B). Sequencing and enzymatic digestion by *Nde*I for F508del and *Xba*I for CFTR KO rats were performed to identify gene‐edited rats (Figure 1C,D). As summarized in Table 1, 9 out of 54 pups and 18 out of 53 pups carried the knockin NdeI and XbaI sequences, respectively. One F508del and two CFTR KO founders (MUKORATs 8.3 and 6.4, referred to as CFTR KO) were crossed with a WT partner and the corresponding mutations were transmitted to the offspring.

**Table 1:** Microinjection data for generation of F508del and CFTR KO rats

Rat model of CF	No. of viable embryos/no. of injected (%)	No. of pups/no. of transferred embryos (%)	No. of NHEJ+ (%)^a^	No. of HDR+ (%)^a^
F508del	171/200 (85.5)	54/171 (31.6)	25^b^ (46.3)	9^c^(16.7)
CFTR KO	322/229 (71)	53/216 (24.5)	26^b^ (49)	18^d^(34)

Abbreviations: CFTR, cystic fibrosis transmembrane conductance regulator; HDR, homology‐directed repair; NHEJ, nonhomologous end joining.

a Calculated from the number of analyzed pups.

b Animals carrying Indels mutations.

c HDR + animals identified by *Nde*I digestion.

d HDR + animals identified by *Xba*1 digestion.

### 3.2 Survival and growth

**Figure 2:**
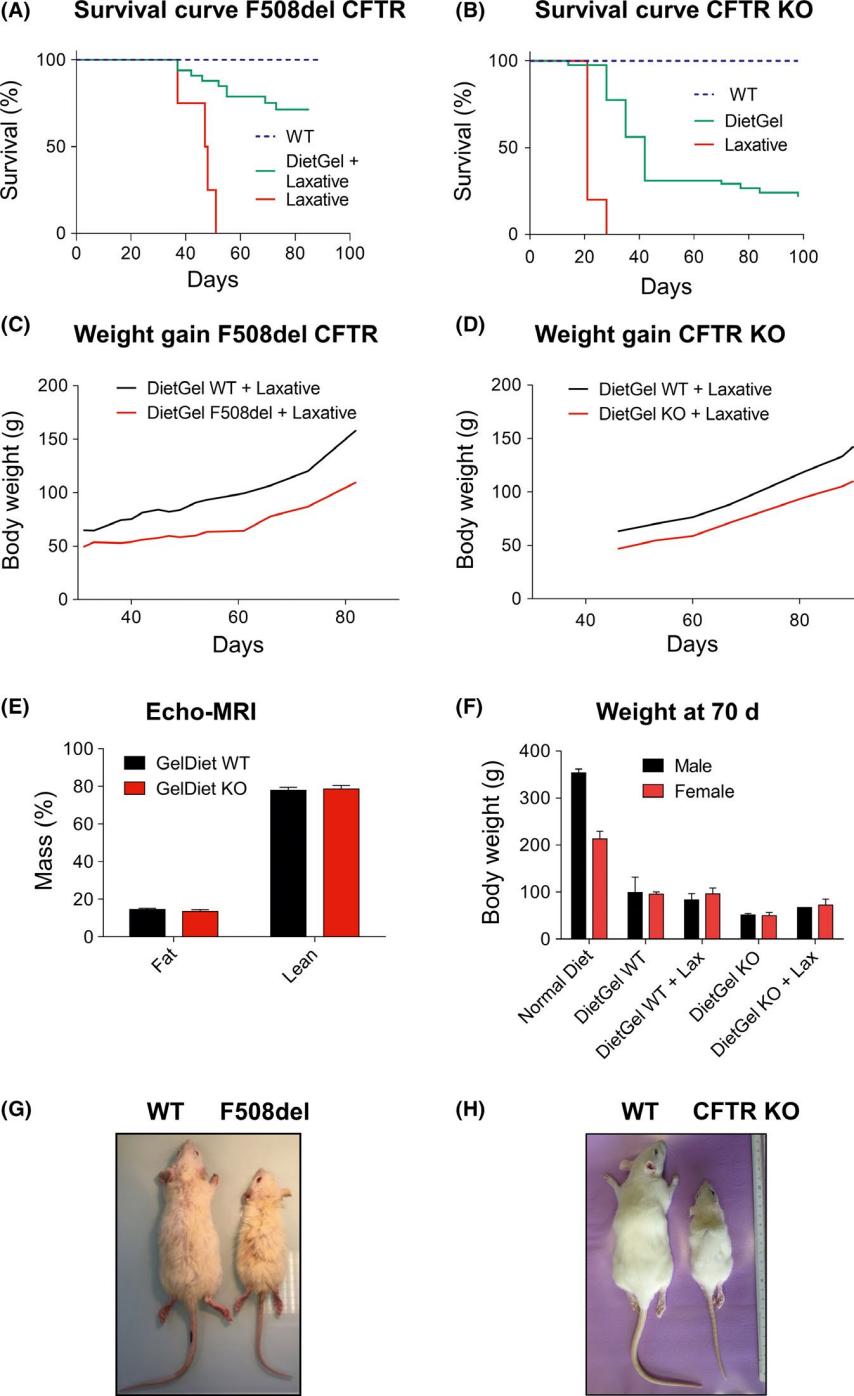
Decreased survival and growth rates of F508del and cystic fibrosis transmembrane conductance regulator (CFTR) KO rats. A, Survival curve of F508del rats receiving DietGel plus laxative (green; N = 33) or laxative alone (red; N = 4) compared to wild‐type (WT) rats (blue; N = 24). B, Survival curve of CFTR KO rats receiving DietGel (green; N = 38) or laxative alone (red; N = 6) compared to WT rats (blue; N = 20). C, Weight gain of F508del rats (red, N = 7) receiving DietGel plus laxative compared to WT rats (black; N = 7). D, Weight curve of CFTR KO rats (N = 4) receiving DietGel plus laxative (red) compared to WT rats (black; N = 5). E and F, Growth of CFTR KO rats fed with DietGel and comparison between male and female animals. Fat/Lean mass evaluated by Echo‐MRI in WT (N = 13) and CFTR KO (N = 11) rats aged 100‐240 d (E). Comparison between male (black) and female (red) rats for weight gain at 70 d of age. WT rats were fed a normal diet (N = 6 males; N = 6 females) or DietGel (N = 8 males; N = 5 females) or DietGel plus laxative (N = 3 males; N = 2 females) (F). G, Three months old WT and F508del rats. H, Four months old WT and CFTR KO rats

F508del and CFTR KO rat models both showed high mortality at weaning that could not be compensated for by the addition of laxative to drinking water (Figure 2A,B). Autopsies performed on F508del and CFTR KO rats revealed intestinal obstruction, which usually occurred at the level of the cecum with a dilated appearance of the small intestine (Figure S1A,B). In surviving CF animals, however, no macroscopic abnormalities of the digestive tract were observed when compared to WT rats (Figure S1C‐F). Replacement of solid food with DietGel + laxative improved survival to 75% for F508del rats in contrast to CFTR KO rats whose survival was only improved to 25% (Figure 2A,B). Weight gain after weaning was decreased in F508del and CFTR KO rats compared with their WT littermate (Figure 2C,D). CFTR KO animals did not display an abnormal fat/lean mass distribution (Figure 2E). Figure 2F illustrates the weight at 70 days of life of male and female CFTR KO and WT rats fed with DietGel + laxative compared to WT rats fed with normal chow. Although WT rats showed reduced weight gain, it had no effect on animal survival. Figure 2G,H show the growth difference for both F508del and CFTR KO rats compared to their respective WT littermates. Altogether, both models exhibited failure to thrive but CFTR KO rats displayed a more severe nutritional and intestinal phenotype when compared to F508del animals.

### 3.3 CFTR‐dependent phenotypes

**Figure 3:**
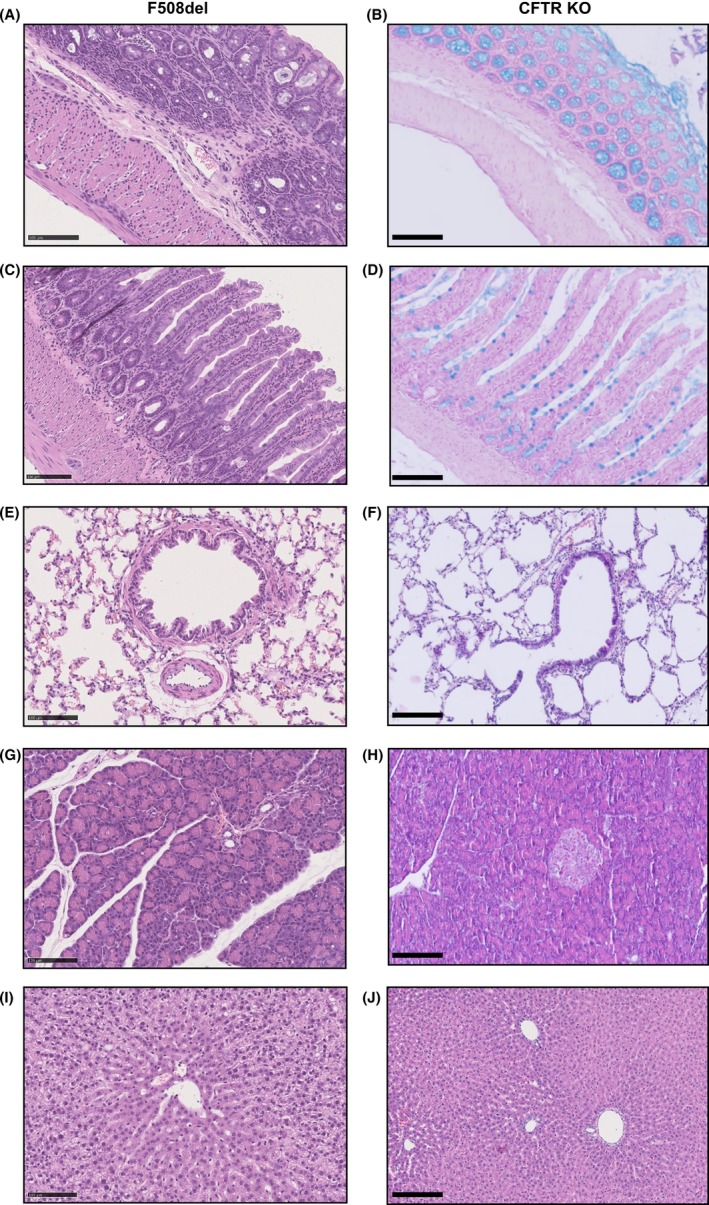
Normal histology of different organs dissected from F508del and cystic fibrosis transmembrane conductance regulator (CFTR) KO rats. Hematoxylin and eosin‐stained sections of colon (A, B), ileum (C, D), lung (E, F), pancreas (G, H), and liver (I, J) from F508del and CFTR KO rats, respectively. All sections from CFTR KO rats were also stained with Alcian blue. Scale bar: 100 µm for F508del rats and 75 µm for CFTR KO rats

Our study reports for the first time the phenotype of F508del rats aged less than 6 months. The histology of colon, ileum, lung, pancreas, and liver of F508del animals showed no obvious histological abnormality nor sign of inflammation on H&E (Figure 3) and PAS (data not shown), as compared to WT tissues (Figure S2). Examination of tracheal sections did not show differences in SMGs between F508del and WT rats (data not shown).

**Figure 4:**
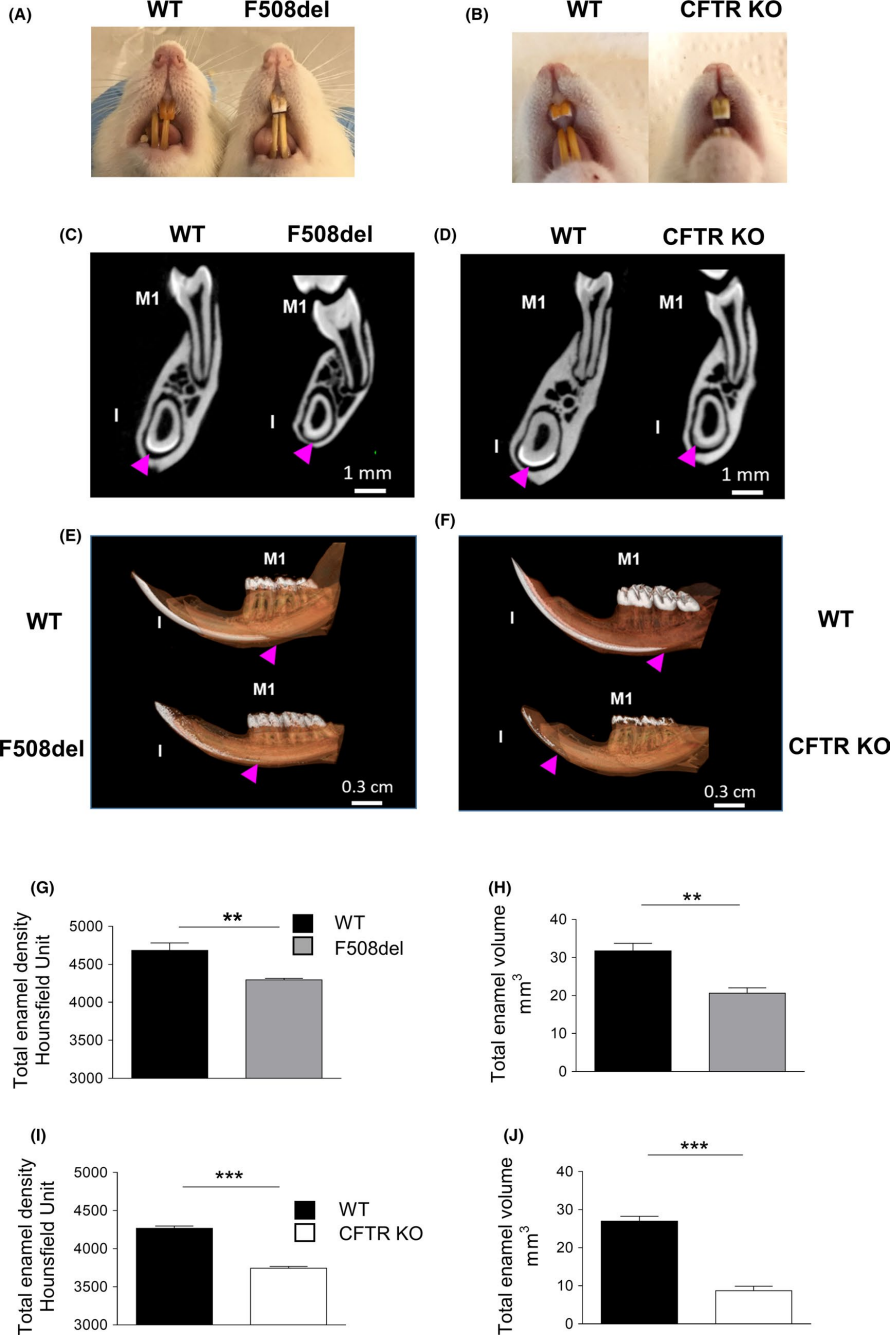
Abnormal tooth enamel phenotype of F508del and cystic fibrosis transmembrane conductance regulator (CFTR) KO rats. A, F508del and (B) CFTR KO rats showed white discolored incisors in contrast to normal yellow wild‐type (WT) rat incisors. C and D, 2D sections and E and F, 3D reconstructed micro‐CT sections (scale bar: 1 mm) showing a delay in enamel mineralization in continually developing incisors (I, arrowhead) in both rat models when compared to matching WT teeth. In WT rats, enamel mineralization was observed under the first molar (M1), whereas it was rarely detected ahead of M1 in F508del and was hardly distinguishable in CFTR KO rats (scale bar: 0.3 cm). G and H, quantitative analysis showed a significantly lower total (molars and incisors) enamel density (G) and volume (H) in F508del rats (grey, N = 8) when compared to matching WT rats (black, N = 4‐6). **P < .01, WT vs F508del rats. (M1) first molar; (I): incisor, Mann‐Whitney test. I and J, abnormal enamel density (I) and volume (J) of CFTR KO rats. Quantitative analysis showed a significantly lower total (molars and incisor) enamel density and volume in CFTR KO (white, N = 8) rats when compared to their WT littermates (black, N = 4‐6). ***P < .001 WT vs CFTR KO rats, Mann‐Whitney test

Dental examination of adult F508del rats showed pronounced discoloration of the continually growing incisors, which appeared white in contrast to the normal yellowish color of WT incisors (Figure 4A). Of note, the enamel of continually growing incisors showed dramatically delayed mineralization; mineralized enamel was observed at the level of the first molar (M1) in WT animals whereas it was detected only weakly ahead of M1 in F508del incisors (Figure 4C,E). Quantitative analysis from high‐resolution micro‐CT of mandible samples showed a lower enamel density and volume in F508del rats when compared to their respective WT littermates (*P* < .01) (Figure 4G,H). CFTR KO rats displayed a phenotype similar to F508del rats except for enamel mineralization, which was hardly distinguishable in incisors at the level of M1 (Figure 4B,D,F,I,J).

**Figure 5:**
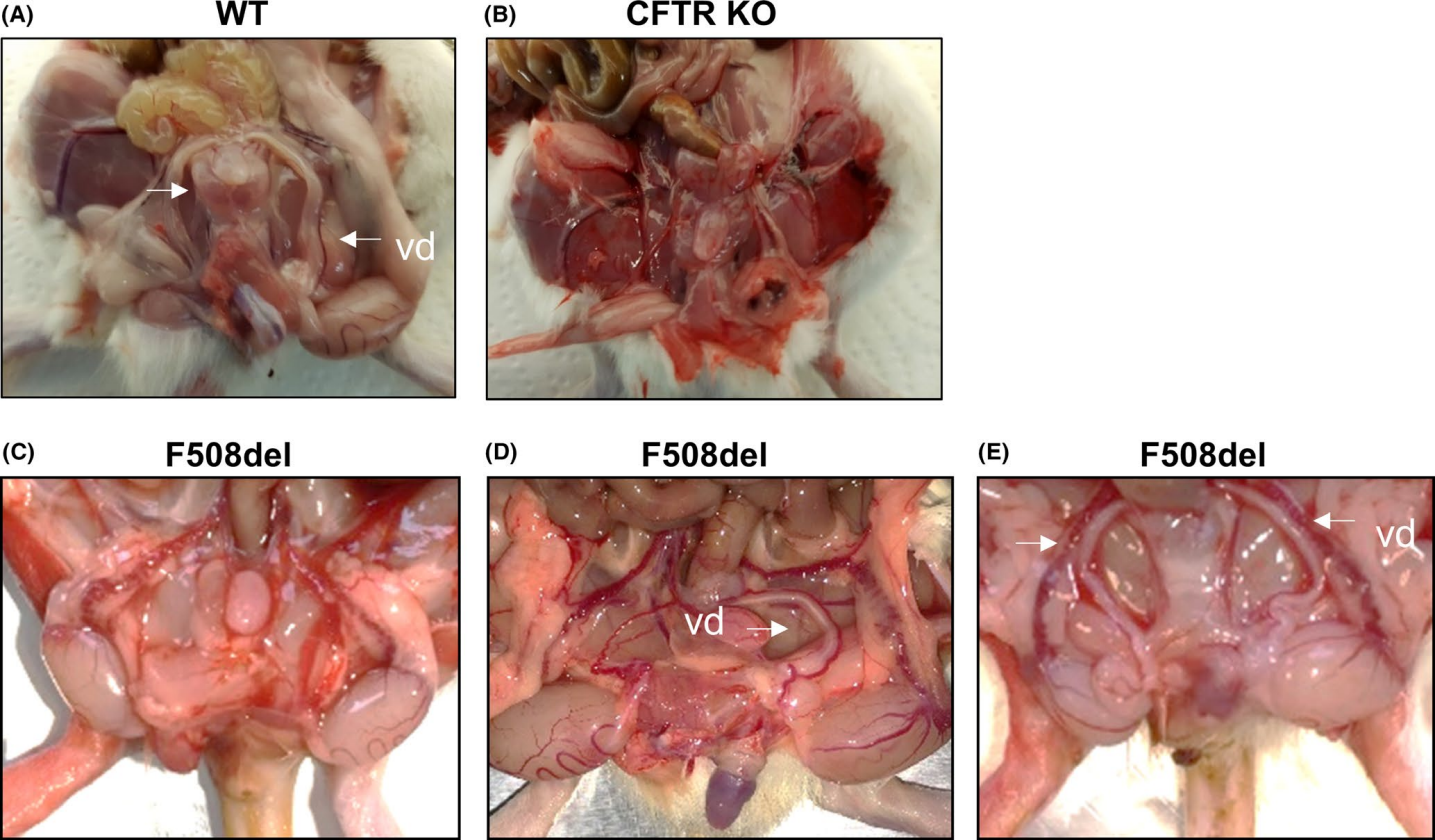
*Vas deferens* abnormality of CFTR KO and F508del male rats. Wild‐type (WT) rats (A) have two *vas deferens* as compared to CFTR KO rats (B) where no *vas deferens* were found. F508del rats had either no *vas deferens* (C) or only one (D) or two (E) *vas deferens*

Cystic fibrosis transmembrane conductance regulator KO males exhibited bilateral agenesis of the *vas deferens* in contrast to F508del males that could have either a normal phenotype or the absence of one or two *vas deferens* (Figure 5).

### 3.4 Nasal potential difference

**Figure 6:**
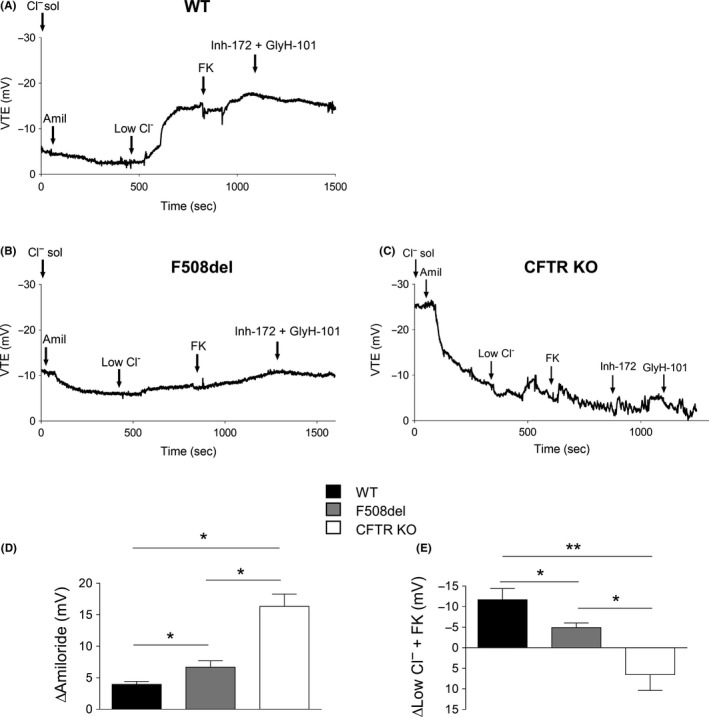
Nasal potential difference measurements in wild‐type (WT), F508del, and cystic fibrosis transmembrane conductance regulator (CFTR) KO rats. Representative transepithelial voltage (*V*_TE_) recordings of WT (A), F508del (B), and CFTR KO (C) rats. Quantification of ∆Amiloride (D) and ∆Low Cl^−^ + FK (E) in WT (black; N = 7), F508del (grey; N = 7), and KO (white; N = 3) rats. *P < .05, **P < .01, Mann‐Whitney test

Nasal potential difference (NPD) was monitored in seven F508del and three CFTR KO rats. As expected, WT controls (Figure 6A, N = 9) displayed robust Cl^−^ secretion, as indicated by the response to low Cl^−^‐containing medium and the application of forskolin, which was partially inhibited by Inh‐172 and GlyH‐101 (Table 2). This response was decreased significantly by 64% (*P* < .05) in F508del rats (Figure 6B,E; Table 2) and was absent in CFTR KO animals (Figure 6C,E; Table 2). Quantification of the NPD changes evoked by amiloride and Low Cl^−^ + FK in F508del and CFTR KO rats is shown in Figure 6D and E, respectively. The defect in Cl^−^ secretion was associated with an increase in Na^+^ transport, as revealed by an increased amiloride response in both F508del and CFTR KO rats (Figure 6D; Table 2). These recordings are consistent with impaired CFTR‐dependent Cl^−^ secretion in the nasal airways of mutant rats.

**Table 2:** NPD parameters for WT, F508del, and CFTR KO rats

NPD (mV)	WT	F508del	CFTR KO	*P* value^a^		
Mean (SEM)				WT/F508del	WT/CFTR KO	F508del/CFTR KO
Baseline	−6.9 (1)	−12.1 (1.2)	−26.5 (2.1)	.006	.01	.02
∆Amiloride	4 (0.5)	6.7 (1.1)	16.4 (1.1)	.02	.01	.02
∆LowCl^−^ + FK	−11.7 (2.7)	−4.9 (1.1)	6.5 (2.2)	.03	.009	.02
∆Inh‐172 + GlyH‐101	1.1 (1)	1.9 (0.7)	4.6 (2.4)	NS	NS	NS

Abbreviations: CFTR, cystic fibrosis transmembrane conductance regulator; FK, forskolin; NPD, nasal potential difference; WT, wild‐type.

a Mann‐Whitney test.

### 3.5 Intestinal bioelectrical measurements

**Figure 7:**
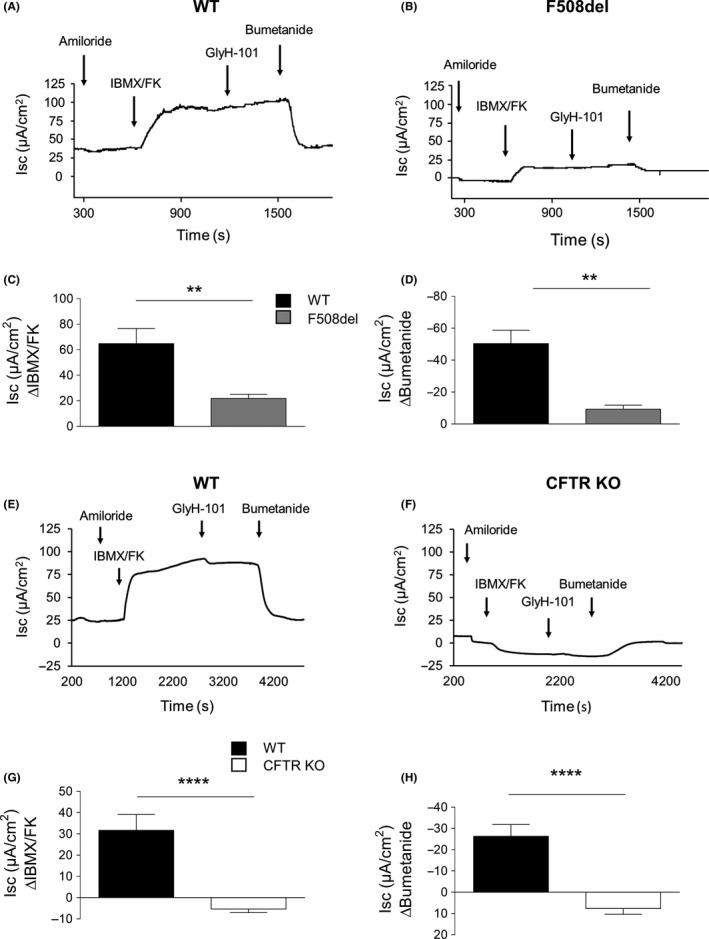
Short‐circuit current (*I*_sc_) abnormalities of F508del and cystic fibrosis transmembrane conductance regulator (CFTR) KO colon mucosa. Representative *I*_sc_ recordings of wild‐type (WT) (A) and F508del (B) colons. Quantification of *I*_sc_ ∆IBMX/FK (C) and ∆Bumetanide (D) in WT (black; N = 6) and F508del (grey; N = 8) rats. Representative Isc recordings of WT (E) and CFTR KO (F) colon mucosa. Quantification of Isc ∆IBMX/FK (G) and ∆Bumetanide (H) in WT (black; N = 12) and CFTR KO (white; N = 7) rats. **P < .01, ****P < .0001, Mann‐Whitney test

Bioelectrical colon tissue properties from WT, F508del, and CFTR KO rats were evaluated for CFTR by short‐circuit current measurements in Ussing chambers (Figure 7; Table 3). WT tissues exhibited a response to IBMX/FK (Figure 7A,C,E,G) and Bumetanide (Figure 7A,D,E,H). These responses were strongly reduced by 66% (*P* < .01) for 3‐isobutyl‐1‐methylxanthine (IBMX)/FK and by 82% (*P* < .01) for Bumetanide in F508del rats (Figure 7B‐D), and were virtually absent in CFTR KO animals (Figure 7F‐H). These data are consistent with residual CFTR conductance in the colonic mucosa of F508del rats, which disappeared in CFTR KO animals.

**Table 3:** Short‐circuit currents parameters on colon of WT, F508del, and CFTR KO rats

*I*_SC_ (µA/cm^2^)	WT	F508del	WT	CFTR KO	*P* value^a^		
Mean (SEM)					WT/F508del	WT/CFTR KO	F508del/CFTR KO
Baseline	8.4 (3.3)	1.1 (2.8)	25.5 (3.1)	0.6 (3.8)	NS	.005	NS
∆Amiloride	−6.3 (1.2)	−5.6 (1.8)	−2.0 (0.4)	−6.1 (1.5)	NS	.02	NS
∆IBMX/FK	64.8 (11.8)	21.8 (3.2)	31.7 (7.4)	−5.3 (2.2)	.001	<.0001	.0003
∆Bumetanide	−50.4 (8.4)	−9.3 (2.5)	−26.3 (5.6)	7.6 (2.8)	.001	<.0001	.0004

Abbreviations: CFTR, cystic fibrosis transmembrane conductance regulator; FK, forskolin; *I*_SC_, short‐circuit currents; WT, wild‐type.

a Mann‐Whitney test.

### 3.6 Primary cultures of nasal airway epithelial cells from F508del rats

**Figure 8:**
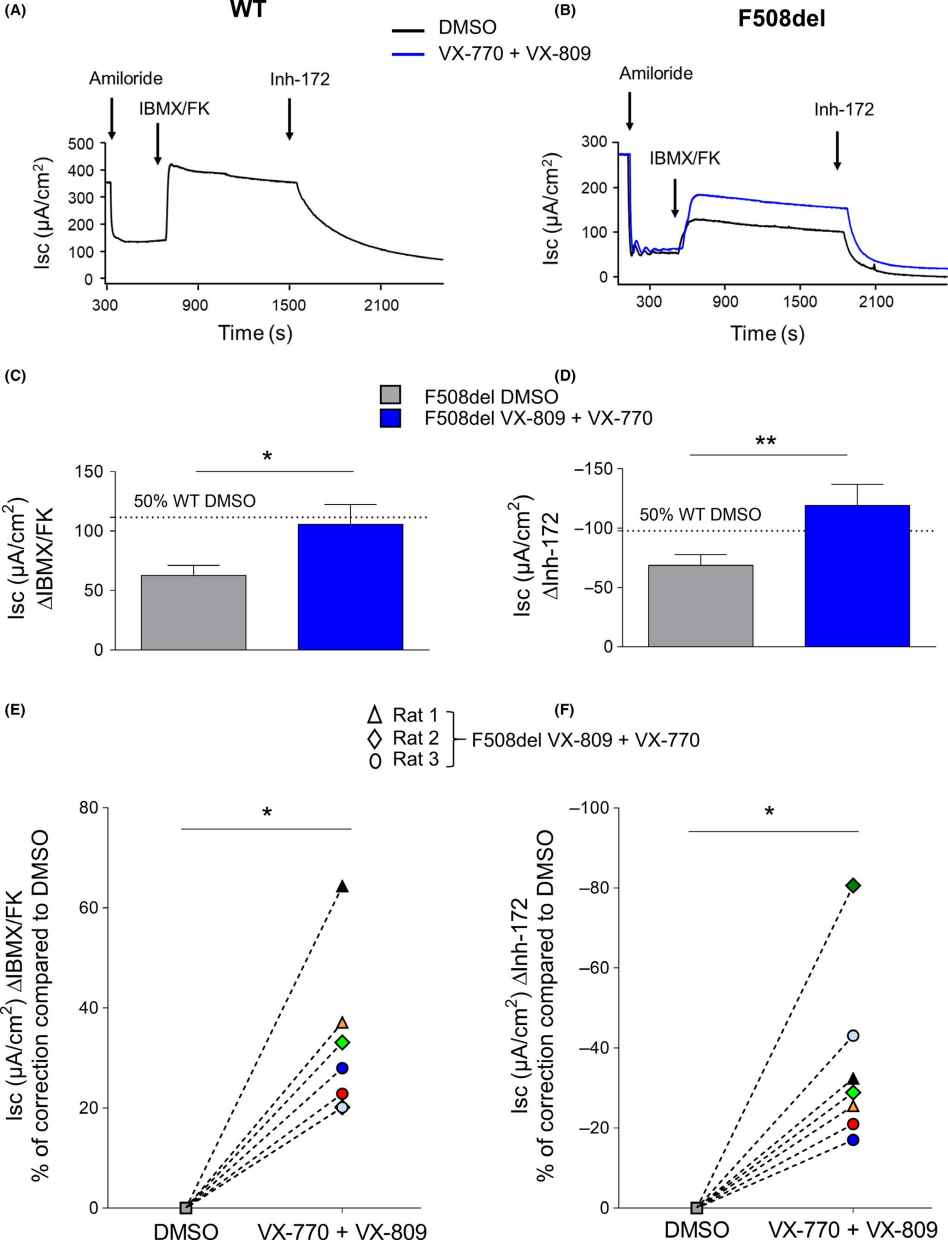
Short‐circuit current (*I*_sc_) abnormality of F508del primary nasal cell cultures and correction by VX‐809 + VX‐770. Representative Isc recordings of wild‐type (WT) (A) and F508del (B) primary nasal cells incubated for 48 h with DMSO (black trace) or VX‐770 (100 nmol/L) + VX‐809 (10 µmol/L) (blue trace). Quantification of *I*_sc_ ∆IBMX/FK (C) and ∆Inh‐172 (D) in F508del primary nasal cells exposed to DMSO (grey; n = 15; N = 4) or VX‐809 + VX‐770 (blue; n = 7; N = 3). The dotted line corresponds to the value of 50% WT DMSO. N represents the number of rats and n represents the number of filters. *P < .05, **P < .01, Mann‐Whitney test. Percentage correction compared to DMSO for Isc ∆IBMX/FK (E) and ∆Inh‐172 (F) in F508del primary nasal cells exposed to DMSO (square grey; n = 7; N = 3) or VX‐809 + VX‐770 (n = 7; N = 3). Each color corresponds to one filter of F508del primary nasal cells exposed to VX‐809 + VX‐770. *P < .05, Wilcoxon test

Measurements of the short‐circuit current were performed on primary nasal cells from WT and F508del rats (Figure 8). WT primary cells treated with Dimethyl Sulfoxide (DMSO) displayed a mean repolarization of 223.1 µA/cm^2^ (±27.5 µA/cm^2^) in response to IBMX/FK, which was totally inhibited by Inh‐172, demonstrating that it was related to CFTR activity (Figure 8A,C,D; Table 4). These responses were decreased by 72% (*P* < .0001) for Forskolin/IBMX and by 65% (*P* < .0001) for Inh‐172 in F508del rat primary nasal cells (Figure 8B‐D; Table 4). No difference was observed between WT cells treated with DMSO or with VX‐809 or VX‐770 (data not shown). In contrast, the combination of VX‐809 + VX‐770 improved CFTR‐dependent Cl^−^ transport in F508del cells as assessed by an 32% increase (*P* < .05) in response to IBMX/FK and 37% (*P* < .05) for Inh‐172 (Figure 8B‐D; Table 4). Although there was some variability in the responses, Figure 8E,F show that all individual changes displayed the same profile. Of note, when VX‐770 or VX‐809 was applied no difference was observed in response to IBMX/FK or Inh‐172 (Table 4).

**Table 4:** Short‐circuit currents parameters of primary WT and F508del primary nasal cells treated with DMSO, VX‐770 + VX‐809, VX‐809, or VX‐770

I_SC_ (µA/cm^2^) Mean (SEM)	WT	F508del				*P* value^a^			
	DMSO	DMSO	VX‐770 + VX‐809	VX‐809	VX‐770	DMSO WT/F508del	DMSO vs VX‐770 + VX‐809	DMSO vs VX‐809	DMSO vs VX‐770
Baseline	301 (24.7)	119 (19.2)	145 (30.5)	55 (63.1)	136 (35.8)	<.0001	NS	NS	NS
∆Amiloride	−157 (18.2)	−90 (16.3)	−106 (24.4)	−53 (38.5)	−105 (27.7)	.005	NS	NS	NS
∆IBMX/FK	223 (27.5)	62 (8.4)	105 (16.9)	92 (21.5)	87 (16.4)	<.0001	.02	NS	NS
∆Inh172	−195 (27.5)	−68 (9.1)	−119 (17.7)	−98 (22.2)	−91 (17.1)	.0001	.008	NS	NS

Abbreviations: FK, forskolin; *I*_SC_, short‐circuit currents; WT, wild‐type.

a Mann‐Whitney test.

## 4 DISCUSSION

This study reports, for the first time, the phenotypic characterization of F508del and CFTR KO rats generated using the Crispr‐Cas9 strategy. Importantly, our results suggest that primary respiratory cells from F508del rats may provide a good model for preclinical testing of CFTR modulators.

Tuggle et al generated a CFTR KO rat model by targeting exon 3 of *Cftr* with zinc‐finger endonuclease^8^ and reported phenotypes similar to those in humans, including growth defects, intestinal obstruction, abnormal dentition, agenesis of *vas deferens*, electrophysiological abnormalities and defects in the structure and mucus of the airways. The phenotype of the CFTR KO rat appeared to be similar to that of the previously published *CFTR* ^−/−^ rat, except for higher mortality by distal intestinal obstruction syndrome and no evidence of crypts dilated with mucus in the ileum. Although the reason for this is unclear, it might be due to a difference in diet (liquid gel in European rat vs solid food in the USA rat) and/or in the sanitary status of the animal facilities. We provide herein the first description of F508del rats, which remarkably reproduced some phenotypic features of human disease. First, they display high mortality without a special diet. Unlike mice that need only laxative to improve survival,^17^ the use of a special gel diet in association with laxative was necessary to reduce mortality, which was mainly related to intestinal obstruction. They displayed a failure to thrive compared to WT controls. The mechanisms of growth retardation are unclear, but like reported by Stalvey et al,^9^ it could result from the abnormal intestinal absorption in association with a decrease in Insulin‐like growth factor‐1 secretion and reduced bone growth. F508del rats exhibited various phenotypes with either none, one, or two *vas deferens*, suggesting that *vas deferens* agenesis may occur postnatally. *Vas deferens* was always absent in CFTR‐deficient rats as reported in this study and previously by Plyler et al,^18^ illustrating a dysgenic and perinatal involution.

Interestingly, both models had abnormal enamel shown by severe discoloration of the continuously growing incisors. These observations are consistent with those previously reported in the *CFTR* ^−/−^ rat model.^8^ Furthermore, as enamel defects are reported in patients with CF,^19^ their presence in the two models introduced here strengthen their validity to investigate the disease pathogenesis. Investigation of the jaws by micro‐CT further revealed a strong decrease in global enamel volume and density in both F508del and CFTR KO rat models and a dramatic delay in mineralization of the continuously growing incisors. This latter observation suggests a direct effect of CF on amylogenesis rather than secondary damage.

Histological examination of F508del and CFTR KO rat lung, pancreas, liver, colon and intestine did not reveal any damage. These results are in accordance with the data found in *CFTR* ^−/−^ rats generated by Tuggle et al, except for ileal tissue which showed epithelial cell sloughing and crypts dilated with mucus in their model.^8^ Moreover, Birket et al showed that in rat airways, CF‐related abnormalities such as SMG hypertrophy and increased mucus viscosity appeared after 6 months.^10^ This suggests that CF‐related disease appears with age and that our rat models were probably too young to exhibit histological alterations. Investigations should be continued in older animals in order to highlight the development of CF.

Our data confirm the abnormal epithelial Na^+^ and Cl^−^ transport that reflects defects in CFTR channel activity. CFTR KO rats exhibited an absence of Cl^−^ secretion in nasal and colon epithelia, in accordance with the results reported previously by Tuggle et al.^8^ F508del rats showed a significant decrease in epithelial response to low Cl^−^‐FK stimulation but Cl^−^ secretion was not totally abolished. F508del rats expressed residual CFTR function, as reported for F508del mice strains (FvB/n; C57BL/6; 129/sv; FvBx129)^20^, ^21^, ^22^, ^23^ and F508del pigs^23^, ^24^ examined for NPD or short‐circuit current measurements.

In addition to NPD and Isc measurements in biopsies, primary cultures of airway epithelial cells provide a useful model for studies of in vitro ion transport^25^ and for testing new CF therapies. We investigated the effects of CFTR VX‐809 corrector and CFTR VX‐770 potentiator alone or in combination on F508del primary cultures of rat nasal epithelial cells. VX‐770 and VX‐809 alone exhibited a trend toward an increase in cAMP‐dependent Cl^−^ transport. Importantly, the combination of VX‐770 and VX‐809 enhanced Isc after IBMX/FK stimulation, an effect that was markedly reduced by Inh‐172. These results are similar to those obtained in humans where VX‐770 or VX‐809 used separately has a limited effect whereas the combination is clinically effective.^26^, ^27^, ^28^ Our results conflict with those published in other cell models of F508del CFTR. In the *Xenopus laevis* oocyte model, Cui et al showed that VX‐770 potentiated mouse CFTR more strongly than human CFTR.^29^ On the other hand, Bose et al recently showed in NIH‐3T3 and CHO cells that VX‐770 did not have any effect on the mouse F508del CFTR Cl^−^ channel whereas it could be corrected by VX‐809.^30^ The difference between these studies might be due to cell model specificity. Altogether, these results show that the F508del CFTR rat provides a valuable model for screening new molecules and predicting their efficacy in humans.

This new F508del rat model appears to be similar to that of F508del *Cftr* mouse by failing to exhibit obvious lung, pancreas, or liver disease. Unlike the CFTR KO rat, it displays milder intestinal phenotype, increased weight gain, reduced congenital bilateral absence of the *vas deferens* and less severe enamel defects. This is possibly due to the residual CFTR function in the respiratory and intestinal epithelium, which interestingly can be enhanced further in ex vivo primary nasal cultures by the combination of VX‐809 and VX‐770. This high residual activity is not observed in most homozygous F508del CF patients and may be due to marked sequence differences between the humans and rodent *CFTR* gene. This may result in an overestimation of the efficacy of CFTR modulators, as well as in a milder phenotype than observed in humans. Clearly, older rats need to be investigated in order to get better insight on whether the CF rat model can contribute to our understanding of mucus stasis in the airways linked to the dysfunction of SMGs. Importantly, this model could be used to study the effect of CFTR modulators in vivo including pharmacokinetics and toxicity of new drugs.

## CONFLICT OF INTERESTS

Dr. Sermet‐Gaudelus reports grants from Vertex Therapeutics, personal fees from Vertex Therapeutics, personal fees from Eloxx, nonfinancial support from PTC Therapeutics, outside the submitted work.

## AUTHOR CONTRIBUTIONS

ED, MB, and JS performed the experiments and analysis, prepared figures, and wrote the manuscript. LG, AH, LV, and DT contributed to some experiments. CU, LT, and IA generated the rat models. LS and JS performed micro‐CT measurements. JPC designed sgRNA sequences. ISG, MC, CHC designed and supervised the research, and wrote the manuscript. All authors reviewed the manuscript.
