# Supplementary figures and images for: Characterization of two rat models of cystic fibrosis—KO and F508del CFTR—Generated by Crispr‐Cas9
Source: Animal Model Exp Med. 2019 Nov 25;2(4):297–311. doi: 10.1002/ame2.12091 (PMC6930998; doi:10.1002/ame2.12091)

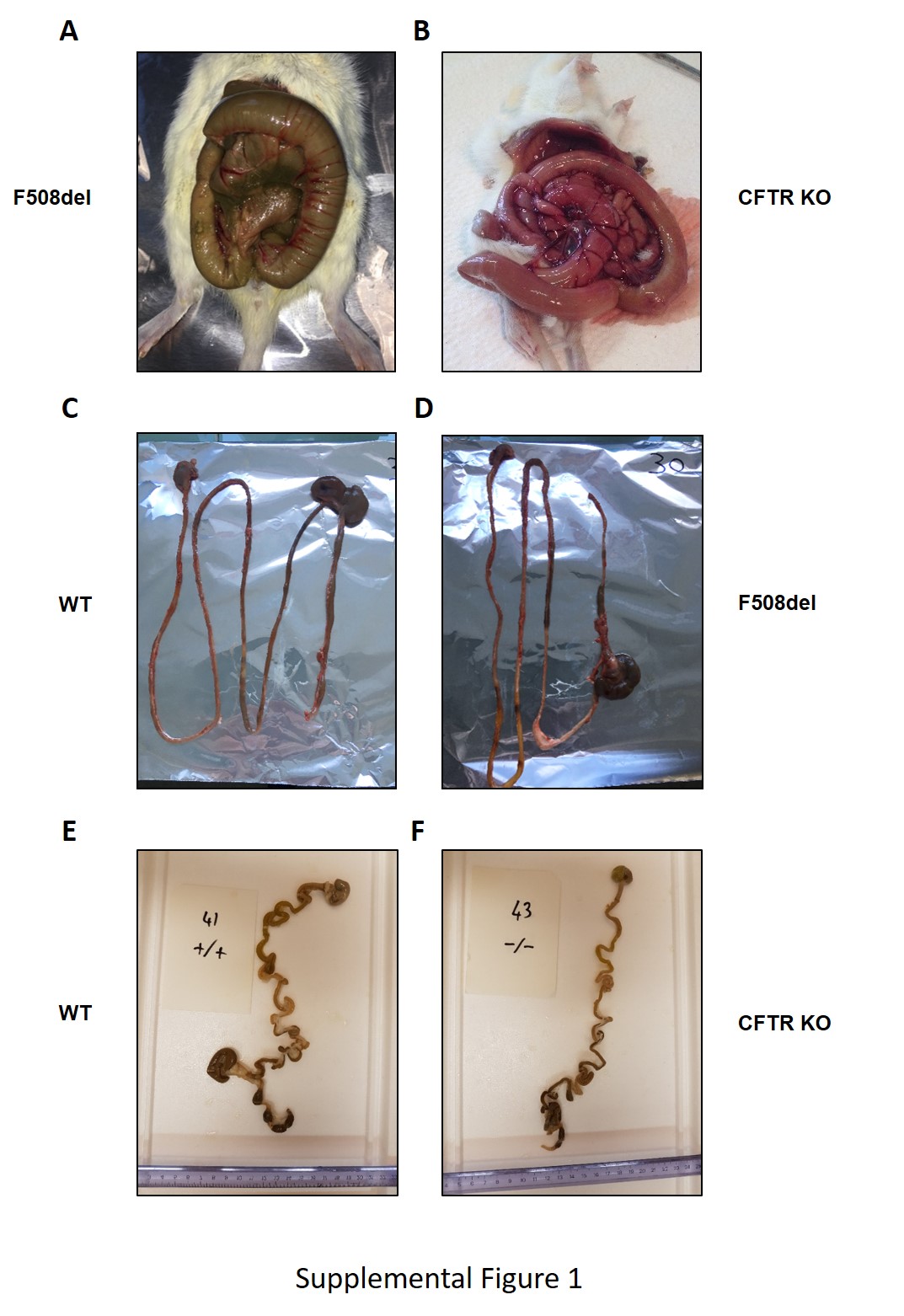

Supplement: Supplementary file 1 [file AME2-2-297-s001.jpg]

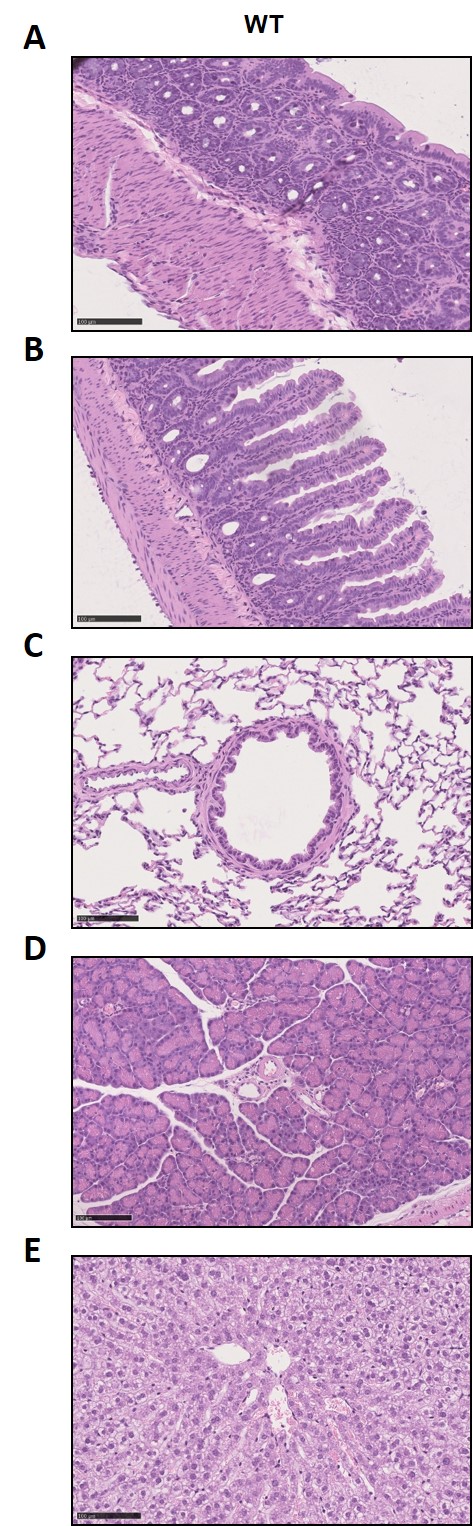

Supplement: Supplementary file 2 [file AME2-2-297-s002.jpg]
