# Supplementary material for: Characterization of two rat models of cystic fibrosis—KO and F508del CFTR—Generated by Crispr‐Cas9
Source: Animal Model Exp Med. 2019 Nov 25;2(4):297–311. doi: 10.1002/ame2.12091 (PMC6930998; doi:10.1002/ame2.12091)
Supplement: Supplementary file 3 [file AME2-2-297-s003.docx]

**Supplementary Materials and Methods**

*Generation of F508del and CFTR KO rats*

For F508del rats, sgRNA was designed, produced and tested *in vitro* in rat C6 cells by T7 endonuclease I mismatch detection by the TACGENE platform (Paris). sgRNA#12 targeting the following sequence, CTTATATCTGTACTCATCAT, was purchased from IDT and was complexed to SpyCas9-3NLS protein (TACGENE) prior to microinjection by the TRIP platform (Nantes, France). The ssODN sequence rCFTRsPS (IDT) was: 5'-G*T*T*TCATTCTCCTCTCAAATTTCTTGGATTATGCCGGGTACTATCAAAGAAAATATCATCGGTGTTTCATATGATGAGTACAGATATAAGAGTGTTGTCAAAGCTTGCCAACTAC*A*G*G-3' (* = phosphorothioate). The NdeI site is underlined (Fig. 1A).

For CFTR KO rats, sgRNA was designed, produced and tested *in vitro* in rat C6 cells by T7 endonuclease I mismatch detection by the GenoCell Edit platform (Nantes, France). sgRNA targeting the following sequence, TTCTTCTGATTCAGCTGACC, was transcribed *in vitro* with the T7 high yield kit (New England Biolabs) and was purified using an EZNA microelute RNA Clean-UP column (OMEGA Biotek) by the TRIP platform (Nantes), and was complexed to SpyCas9-3NLS mRNA (TACGENE) prior to microinjection. The **ssODN** sequence (TACGENE) was:

5’TGGACCACACCAATTTTGAGAAAAGGGTACAGACACCACTTGGAGCTGTCAGACATATACCAAGCCCCTTCTAGATTCAGCTGACCACCTGTCTGAAAAGCTAGAAAGG-3’. The XbaI site is underlined (Fig. 1B).

Fertilized one-cell stage embryos were collected from donor female rats (Sprague Dawley/Crl; Charles River, l’Arbresle, France) for subsequent microinjection by the TRIP platform (Nantes, France) using a previously published procedure (Ménoret *et al., Sci Rep; 2015*). Briefly, for F508del, a mixture of Cas9 protein (3 µM), sgRNA (150 ng/µl) and ssODN rCFTRsPS (15 ng/μl) or for CFTR KO, a mixture of Cas9 mRNA (50 ng/µl), sgRNA (10 ng/µl) and ssODN (15 ng/µl) was microinjected into the male pronucleus of fertilized one-cell stage embryos. Microinjected zygotes were maintained under 5% CO_2_ at 37°C for 2 h. Surviving embryos were implanted on the same day in the oviduct of pseudopregnant females (0.5 dpc) and allowed to develop to full term. One F508del and two KO lines were obtained and referred to as MUKORAT 8.3 and 6.4, respectively. Genotyping was performed using methods based in PCR and microcapillary electrophoreses already described in detail.

All animal care and procedures performed in this study were approved by the Animal Experimentation Ethics Committee of the Pays de la Loire region, France, in accordance with the guidelines of the French National Research Council for the Care and Use of Laboratory Animals (Permit Numbers: CEEA-PdL-2015-692). All animal care and procedures performed in the Paris facility were approved by the Animal Experimentation Ethics Committee of Paris Descartes University and were registered with ministerial numbers APAFiS # 9462 and APAFiS # 13755. All animal studies performed in Geneva were approved by the Swiss Federal Veterinary Office and were in accordance with established Swiss guidelines and regulations.

*Animal husbandry of F508del rats*

Expansion, embryo conservation and decontamination of F508del CFTR heterozygous rats were performed by Janvier Labs (Saint Berthevin, France). Seven heterozygous rats (three males: M1, M2, M3, and four females: F1, F2, F3, F4) were sent to the facility of the Faculty of Pharmacy in Paris. Rats were housed in specific pathogen-free conditions and bred in the following scheme: (M1 x F1, M2 x F2, M3 x F3, M3 x F4). When the females were identified as pregnant, male rats were removed. Twelve days after pupping, chows were removed and replaced by DietGel 31M and 6% of osmotic laxative (PEG 3350 - KleanPrep) was added to the drinking water. Because a delay in weight gain occurred in WT first littermates, DietGel boost was added to 31M (1:1) for the next WT and CF littermates to improve weight gain. Heterozygous rats showed a normal phenotype. Offspring were identified with earrings about 15 days after birth and tail biopsies were withdrawn for genotyping. Animals were used at the age of 8-14 weeks.

*Animal husbandry of CFTR KO rats*

Heterozygous *CFTR^+/-^* rats were housed in Geneva under conventional housing and bred to generate CFTR KO animals. When the females were identified as pregnant, male rats were removed. High mortality was observed for both 8.3 and 6.4 MUKORATs after weaning due to severe intestinal obstruction that could not be compensated for by iso-osmotic laxative (Transipeg; Mundi Pharma, Basel, Switzerland) added to the drinking water. To reduce mortality, CFTR^-/-^ and CFTR^+/+^ littermates were fed with a liquid diet composed of DietGel 31M Clear H_2_O (ssniff-Spezialdiäten GmbH; Soest, Germany). Due to the malnutrition state of CFTR KO rats, our veterinary authorities required us to euthanize the CFTR^-/-^ rats when they fell below 15% of the weight of their control littermates. Thus, the diet was completed progressively by feeding *CFTR^-/-^* and *CFTR^+/+^* littermates with ground chow mixed with laxative-containing water (DietGel + laxative). No differences in terms of survival or weight gain were observed between 8.3 and 6.4 MUKORATs so that data obtained from these animals (referred to as CFTR KO rats) were pooled. Heterozygous rats showed a normal phenotype. CFTR KO embryos of both lines were cryopreserved (Charles River, l’Arbresles, France). Animals were used at the age of 8-14 weeks.

*Genotyping*

Tail or ear biopsies from 8- to 15-day-old rats were digested and the *CFTR* DNA region targeted by CRISPR nuclease was amplified by PCR. To detect gene edition or knock in, specific CFTR primers were used (see below). Mutations were analysed by NdeI for F508del or XbaI digestion for CFTR KO 8.3 and direct sequencing of PCR products (Fasteris SA, Plan-les-Ouates, Switzerland) for CFTR KO 6.4 animals (Fig. 1C, D).

PCR primers for F508del rats were:

CFTR-n2Fw: 5’-GTGTTGCCCAGGTCTCTTTCAT-3’

CFTR-n2Rv: 5’-GTCTAGCCATCCTCCCATTCCT-3’

PCR product size: 451 bp (WT)

Digestion NdeI (KI): 202 bp + 246 bp

PCR primers for CFTR KO rats were:

rCFTR-Ex3-Up: 5’-GCAAAGGCATCGGTTTAGAG-3’

rCFTR-Ex3-Lo: 5’-GGATTTTCCCTACTGCTGGA-3’

PCR product size: 629 bp (WT)

Digestion XbaI (KI): 378 bp + 249 bp

*Dental characterization*

Mandibles were scanned using a high-resolution X-ray micro-CT device (Quantum FX Caliper; Life Sciences, Perkin Elmer, Waltham, MA) hosted by the PIV platform, EA2496, Paris Descartes University. 3D acquisitions were performed using an X-ray tube voltage of 90 kV and a tube current of 160 μA with a field of view 20 × 20 mm. Full 3D high-resolution raw data were obtained by rotating both the X-ray source and the panel detector 360° around the sample (scanning time of 2 min). Projections were reconstructed by Rigaku software in image blocks of 512 × 512 × 512 voxels with an anisotropic voxel size of 40 μm. Data were stored in Dicom frame. Total enamel volume and density were measured using Analyse 11.0 software (Biomedical Imaging Resource; Mayo Clinic, Rochester, MN). OsiriX Pro imaging software (v5.8.5, distributed under LGPL license, Dr A. Rosset, Geneva, Switzerland) was used from the DICOM stack to reconstruct images for 3D volume and 2D section rendering. Binary thresholds were applied to isolate the enamel from the surrounding tissue.

*Nasal potential difference*

Rats were anaesthetised by intraperitoneal injection of ketamine (133 mg/kg, Imalgene 1000; Merial, France) and xylazine (13.3 mg/kg, 2% Rompun; BayerPharma, France). The exploring electrode was connected to a double-lumen catheter inserted into the nostril to a depth of 5 mm. Cl^-^ solution (140 mM NaCl, 6 mM KCl, 10 mM HEPES, 10 mM glucose, 1 mM MgCl_2_, 2 mM CaCl_2,_ pH adjusted to 7.4) was perfused continuously, at a rate of 0.25 ml/h, into one lumen of the catheter. The second lumen of the catheter was perfused with the five following solutions at a flow rate of 2 ml/h: (i) Cl^-^ solution for baseline measurement; (ii) Cl^-^ solution with 100 µM of amiloride (Sigma-Aldrich, USA, to block ENaC Na^+^ absorption; (iii) low-Cl^-^ solution (140 mM sodium gluconate, 6 mM potassium gluconate, 10 mM HEPES, 10 mM glucose, 1 mM MgCl_2_, 2 mM calcium gluconate_,_ pH adjusted to 7.4) with amiloride to drive Cl^-^ secretion; (iv) low-Cl^-^/amiloride solution with 10 µM forskolin (Sigma-Aldrich, USA) to activate CFTR; (v) low-Cl^-^/amiloride/forskolin solution with 5 µM CFTR_inh_172 (Inh-172) and 10 µM GlyH-101 to specifically inhibit CFTR activity. Each change of solution was performed after the electrical potential reached stability for at least 1 min of recording. Transepithelial potential was recorded using an averaging analogue to digital converter supplied by Logan Research Ltd (Rochester, Kent, UK). Mean values for the last 10 sec of the recordings were analysed.

*Colon short-circuit currents*

Rat distal colon segments of approximately 5 mm in length and opened longitudinally were mounted in Ussing chambers (slider P2407C, 0.018 cm^2^ for F508del rats and P2305, 0.49 cm^2^ for CFTR KO rats). The transepithelial potential difference was voltage-clamped at zero and the resulting Isc was recorded using an EVC4000 Precision V/I Clamp (World Precision Instruments) for F508del tissues or a VCC MC6 amplifier (Physiological Instruments) for CFTR KO tissues. Data were acquired using the interface ADINSTRUMENTS Power Lab and the LabChart 8 Reader or the interface DI-720 (DataQ Instruments) and Acquire & Analyse software 2.3 (Physiological Instruments).

Basal and apical chambers were filled with Krebs buffer (115.5 mM NaCl, 25 mM NaHCO_3_, 2.4 mM K_2_HPO_4_, 0.4 mM KH_2_PO_4_, 1.2 mM CaCl_2_-2H_2_O, 1.2 mM MgCl_2_-6H_2_O, 10 mM glucose and sometimes containing 10 µM indomethacin, and gassed with 95% O_2_, 5% CO_2_ at 37°C. The transepithelial potential difference was voltage-clamped at zero and the resulting Isc was recorded using a V/I Clamp. Epithelial sodium channel (ENaC)-mediated currents were blocked by the addition of 100 µM amiloride at the apical side. CFTR-mediated currents were stimulated by the addition of a cocktail of forskolin (10 µM) and IBMX (100 µM) added to both chambers and inhibited by bumetanide (100 µM) in the basal side to inhibit Na^+^/K^+^/2Cl^-^ co-transport. GlyH-101 (20 µM) was added in the apical side to inhibit CFTR activity.

*Primary nasal cell cultures*

Shortly after euthanasia, the nasal cavity was dissected, treated with a solution containing DTT (5 mM), rinsed with PBS plus antibiotics (Penicillin-Streptomycin (100 µg/ml), Piperacillin-Tazobactam (10 µg/ml), Amphotericin (2.5 µg/ml) and Colimycin (16 µg/ml) and incubated overnight at 4°C in pronase (0.1 mg/ml). The mucosa was scraped with a scalpel to detach epithelial cells in DMEM/F12 medium containing 10% FBS to neutralise the pronase. The cell suspension was filtered through a stainless steel strainer, centrifuged (524 g, 5 min, 4°C) and exposed to trypsin. After centrifugation, the pellet was suspended in DMEM/F12/FBS 10%, supplemented withhydrocortisone (0.2 µM), insulin (5 µg/ml), epinephrine (0.5 µg/ml), EGF (10 ng/ml), Y-27632 (10 µM) and antibiotics). Cells were then seeded in 75-cm² flasks coated with collagen for expansion. Cells were collected by trypsinisation and seeded on porous filter (0.33 cm²; Transwell Corning) coated with collagen at a density of 350’000 cells/filter. After 2 days, the apical medium was removed and the cells were cultured at an air-liquid interface for 20‒30 days with basal medium (DMEM/F12) containing 2% Ultroser G and antibiotics.

Before short-circuit current measurements, primary cells were treated for 48 h on the basal side with DMSO (0.1%), VX-770 (100 nM) or VX-809 (10 µM), or a combination of both.

*Primary nasal cell short-circuit currents*

Cells were bathed in Ringer’s solution and a chloride concentration gradient was applied across the epithelium. Basal Ringer’s solution was composed of 145 mM NaCl, 3.3 mM K_2_HPO_4_, 10 mM HEPES, 10 mM D-glucose, 1.2 mM MgCl_2_ and 1.2 mM CaCl_2_. Apical Ringer’s was composed of 145 mM Na-gluconate, 3.3 mM K_2_HPO_4_, 10 mM HEPES, 10 mM D-glucose, 1.2 mM MgCl_2_ and 1.2 mM CaCl_2_. Each solution also contained 10 µM indomethacin and was gassed with 95% O_2,_ 5% CO_2_ at 37°C.

The transepithelial potential difference was voltage-clamped at zero and the resulting Isc was recorded using an EVC4000 Precision V/I Clamp (World Precision Instruments). Data were acquired using the interface ADINSTRUMENTS Power Lab and the LabChart 8 Reader. Epithelial sodium channel (ENaC)-mediated currents were blocked by the addition of 100 µM amiloride in the apical side. CFTR-mediated currents were stimulated by the addition of a cocktail of forskolin (10 µM) and IBMX (100 µM) added to both chambers and specifically inhibited by the addition of Inh-172 (5 µM) in the apical side.
