# Supplementary material for: Characterization of two rat models of cystic fibrosis—KO and F508del CFTR—Generated by Crispr‐Cas9
Source: Animal Model Exp Med. 2019 Nov 25;2(4):297–311. doi: 10.1002/ame2.12091 (PMC6930998; doi:10.1002/ame2.12091)
Supplement: Supplementary file 4 [file AME2-2-297-s004.docx]

**Figure S1:** Intestinal abnormality in F508del and CFTR KO rats. Intestinal obstruction in F508del **(A)** and CFTR KO **(B)** rats that died prematurely. Normal intestine in surviving WT **(C)** and F508del **(D)** rats. Normal intestine in surviving WT **(E)** and CFTR KO **(F)** rats.

**Figure S2:** Normal histology of different organs dissected from WT rats. H&E stained sections of colon **(A)**, ileum **(B)**, lung **(C)**, pancreas **(D)** and liver **(E)** from WT rats. Scale bar: 100 µm.
